# The Introduction of Entropy and Information Methods to Ecology by Ramon Margalef

**DOI:** 10.3390/e21080794

**Published:** 2019-08-15

**Authors:** William B Sherwin, Narcis Prat i Fornells

**Affiliations:** 1 Evolution & Ecology Research Centre, School of Biological Earth and Environmental Science, UNSW Sydney, Sydney NSW 2052, Australia; 2 Secció Ecologia, Departament de Biologia, Evolución, Ecologia & Ciències Ambiamentales, Facultat de Biologia, Universitat de Barcelona, Diagonal 643, 08028 Barcelona, Spain

**Keywords:** ecology, evolution, succession, alpha diversity, beta diversity, Shannon, entropy, information, Margalef

## Abstract

In ecology and evolution, entropic methods are now used widely and increasingly frequently. Their use can be traced back to Ramon Margalef’s first attempt 70 years ago to use log-series to quantify ecological diversity, including searching for ecologically meaningful groupings within a large assemblage, which we now call the gamma level. The same year, Shannon and Weaver published a generally accessible form of Shannon’s work on information theory, including the measure that we now call Shannon–Wiener entropy. Margalef seized on that measure and soon proposed that ecologists should use the Shannon–Weiner index to evaluate diversity, including assessing local (alpha) diversity and differentiation between localities (beta). He also discussed relating this measure to environmental variables and ecosystem processes such as succession. Over the subsequent decades, he enthusiastically expanded upon his initial suggestions. Finally, 2019 also would have been Margalef’s 100th birthday.

## 1. Margalef’s Introduction of Entropy and Information Theory to Ecology

The year 2019 is very important for the use of entropic methods in biodiversity. It marks the anniversaries of some important milestones in the life of Ramon Margalef, who first introduced ecologists to entropic methods. This year is the 100th anniversary of his birth (16 May 1919) and the 70th anniversary of his Licentiatura de Ciencias Naturales (despite the professors wanting to fail him due to poor attendance) [1]. This was quickly followed by his Ph.D. only two years later. His subsequent career established him as both a superb naturalist and a preeminent theoretical ecologist. It is fitting that someone who was awarded so many scientific prizes [1] should now be memorialized by an extremely important international prize for ecology, the “Premi Ramon Margalef D’Ecologia” [2]. Indeed, the use of entropy/information methods in evolution and ecology has flourished: the journal *Entropy* contains many examples, including in at least 13 of 43 biological special issues, with 6 of those 13 active in 2019 [3]. This paper will outline some of Margalef’s pioneering work and then trace its consequences for the use of entropy and information theory in biodiversity studies.

The year 2019 also marks the 70th anniversary of Margalef’s first attempt to use a logarithmic approach to partition biodiversity within and between groups [4], something that ecologists and geneticists are still developing nowadays [5,6]. In the 1949 paper, Margalef concluded that, for his phytoplankton dataset, Fisher’s log-series parameter (in Table 9 of Reference [7]) was better than Preston’s log-normal [8]. Moreover, in 1949, when he was only just finishing his undergraduate degree, Margalef was already doing many things that we are still trying to deal with properly in biodiversity. He found a way to obtain inferences that were not dependent on sample size, showing that the log-series parameter was independent of sample size [4] (Figure 3), unlike the log-normal [8] (p. 272). Here, and throughout his life, he was viewing the world in the three ways used by all good scientists: actual values (sometimes called “ontic”, and often unknowable), the measures of those values (sometimes called “estimated” or “epistemic”), and the forecasts from models of the underlying system (which can be tested against the estimates). In this paper, we indicate where Margalef explicitly distinguished between these.

In the 1949 paper, Margalef was also seeking methods that appropriately considered species all the way from rare to common. Additionally, he was incorporating functions into his diversity investigations, showing that the log-series parameters responded to whether individuals were clustered on some environmental gradient [4] (Figure 3). Note that Fisher called his logseries parameter “alpha”, but in this article, we reserve “alpha” for another use: any measure of diversity that is calculated for a single location, as defined by Whittaker [9]. Margalef was also searching for a method to characterize diversity between locations, later called “beta” [9], with Margalef attempting “to determine statistically whether or not there was a discontinuity in the floristic composition of the communities … even if the characteristics of the environment were distributed according to a continuous gradient” [4] (p. 67, translation). In this paper, Margalef used two of his datasets on freshwater algae in northeast Spain (12,484 individuals, 653 species).

The year 2019 is also the 70th anniversary of the first presentation to the wider world of Shannon and Weiner’s formula for the entropic content of information [10]:

(1) H1=−∑i=1Spilnpi,

where *p_i_* is the proportional abundance of the *i*th type of symbol (e.g., a letter or a word) in a message of *S* different symbols. In this article, unless stated otherwise, we use “information” or “entropy” interchangeably to refer to H1, avoiding many other interpretations of each word [10,11].

Margalef did not immediately use the Shannon index. His next attempt at a diversity index came in 1951 when he was still searching for an estimate that was independent of sample size [12]. Gleason had shown that in one dataset, the number of species, *S*, was linearly related to the logarithm of the area sampled [13]. Margalef reasoned that if the larger area sampled resulted in a larger sample of individuals (*N*), then a diversity expression that was independent of sample size would be

(2) d=(S)/ln(N+1).

This is also sometimes given as d=(S−1)/ln(N+1), and it is now called the “Margalef diversity index” and is included in many packages despite Margalef’s opinion that this is a “sin of youth” (N.Prat. pers. comm.). As *N* and *S* approach very large values, both of these formulae approach Fisher’s log-series parameter (E. Marcon, pers. comm.). Note that neither Margalef’s nor Fisher’s formulas incorporate abundance, so they weight rare and common species equally. As usual, Margalef was dissatisfied with what he had produced, writing the following: “The regularity of the relationships between presence and abundance in the sense of phytosociologists needs more statistical studies” [12] (translation).

Margalef recognized that any measure based only on the number of different species would represent merely a single aspect of diversity, so he next tried to include the relative abundance of each species by calculating 1/lnr, where *r* is the (geometric) ratio of abundances of successively more abundant species [14]. He compared this to two other indices: his own “*d*” and the log-series parameter of Fisher et al. [7]. Margalef showed that for data for Tintinidae off the coast of Castellón (northeast Spain), the three measures gave the same ranking, unless the total number of species sampled was very small. He pointed out however, that relying upon the geometric ratio was not ideal, because the abundance of each species would be determined by its own balance of births, deaths, immigration, and emigration.

In 1956, Margalef had a two-month trip to North America, which was very successful, including a talk on “temporal succession and spatial heterogeneity in natural phytoplankton” at a meeting in California organized by the Scripps Institute of Oceanography and the Office of Naval Research. In this talk, he touched on applying concepts of information theory to the structure and dynamics of communities of organisms. This idea immediately caught the interest of the audience and gave Margalef the necessary momentum to write a paper in which he moved one step closer to presenting Shannon’s formula to ecologists. In 1956, he was explicitly expressing diversity as information and entropy in datasets of marine phytoplankton from Vigo and Castellón [15]. Here, he used the Brioullin formula to develop a diversity index that simultaneously included the abundance of each species, which was also likely to have relatively little association with sample size:

(3) $$Average\;Entropy\;per\;Individual=B=\frac{1}{N}ln\frac{N!}{N_{1}\ldots N_{i}\ldots N_{S}},$$

where $N_{i}$ is the number of individuals of species *i* in the sample, and *N* is the total of all individuals in the *S* species. Thus, this formula includes both the number of species *S* and their abundances $N_{i}$, as Margalef wished. Margalef noted that a similar formula had been used to express the diversity of amino acids in a protein [16] and pointed out that information theory was particularly useful for generalizing from one scientific field to another. However, he did not show the approximate equivalence of Equations (1) and (3) or cite Shannon and Weaver [10], although he did cite Wiener’s earlier book [17].

In the 1956 paper, Margalef was also concerned with diversity change over time and space and appears to have invented by himself the equation that we now call “mutual information”. His equation for spatial differentiation was

(4) $$Measure\;of\;heterogeneity=\frac{B_{pooled}-\left(B_{1}+B_{2}\right)/2}{L},$$

where $B_{1}\;\mathrm{and}\;B_{2}$ are the values of Equation (3) for two areas, 1 and 2; $B_{pooled}$ is the value for pooled data from those areas; and *L* is the distance separating the two localities. If one uses Equation (1) instead of Equation (3) to calculate $B_{1},\;B_{2}$, and $B_{pooled}$, the top part of this equation is identical to what we now call mutual information, the fundamental Shannon-based measure of diversity due to differences between localities, as formulated in Box 2 of Reference [5]. Margalef did not use the name “mutual information”, because that concept and its name were still developing at the time [18]. For example, when Shannon first published the mutual information formula in his classic paper, he named it “rate of transmission” [19] (p. 21).

Finally, in 1957, Margalef was the first to propose using Shannon’s formula (Equation (1)) in biodiversity studies, where *p_i_* would be the proportional abundance of the *i*th species in a community of *S* different species [20]. He showed that Equations (1) and (3) were equivalent unless the sample size was small. He used his marine phytoplankton data from the Mediterranean and Atlantic coasts of Spain. This publication was in Spanish, but in those days, scientists were expected to read the literature in all languages, often being required as undergraduates to learn one other “scientific” language. The paper was regarded as so important that it was republished a year later in English, despite the fact that scientific journals usually resist republication [21]. As was customary, he went far beyond simply characterizing diversity at one location. He also discussed an analysis of underlying ecological processes, temporal change, and spatial structure (repeating Equation (4)).

More details of Margalef’s life and work may be found in References [1,22,23,24]. This review focuses on his introduction of information/entropy into ecology, noting that his use of these theories was driven by his fascination with highly diverse ecosystems such as plankton (algae, zooplankton), the energetic basis of this diversity, and how this diversity changed through space and time. He was a prodigious publisher, and by the end of 1949, this very junior scientist already had 68 publications upon which he drew for his theoretical work. His breadth of publication expanded to include marine environments as he became more established. By 1952, Margalef was a permanent researcher in the Instituto des Ciencias del Mar, rising to director from 1965 to 1967, then to Professor of Ecology (1967 to 1996), then to Emeritus Professor (1996–2002).

## 2. Shannon Entropy and Information: Patterns in Ecology

From Margalef’s 1957 paper onwards, Shannon’s formula (Equation (1)) went on to become the most frequently used abundance-sensitive biodiversity measure for species in ecosystems [25]. Krebs and Pielou [26,27] acknowledged Margalef as the first to suggest the use of this formula. Shannon’s formula (Equation (1)) has gone on to many different uses in ecology, including being based on relative biomass rather than on relative abundance [28]. Readers should note that Margalef often used the symbol D for the formula in Equation (1) [29] (p. 18), but this is now often called H1, in line with information theory, whereas D or D1 is reserved for the transformation of H into a scale of “effective numbers”, i.e., the number of equally frequent species that would be needed to give the value of H1 that is derived from an assemblage of species with unequal frequencies [5,30].

Shannon’s Equation (1) has many useful properties in ecology. This equation is used to answer the question, “ ‘How difficult would it be to predict correctly the species of the next individual collected?’ This is the same problem faced by communications engineers interested in predicting correctly the name of the next letter in a message.” [27] (p. 506). This is clearly a diversity measure: if there are many equally frequent species, we will have poor predictive ability, whereas if there are only a few species, or a few very common ones, then it will be relatively easy to make this prediction, and the Shannon–Wiener measure quantifies this predictive ability. Moreover, Shannon satisfies some of the important properties of a diversity measure, especially the following:

- That H1 has its maximum value when the species abundances are even;
- That for two even communities, the one with more species has a higher H1;
- That H1 is completely additive in a hierarchy, such as in local areas within a larger area within a continent [31] (p. 291) [5,32].

These and other properties have led to the enormous importance of the Shannon formula, H1, in ecology, but it is important to note that other entropy-based diversity measures are also important, especially H0=S−1 (one less than the total number of species) and H2=1−∑i=1Spi2 (the chance that two randomly drawn individuals belong to different species). When transformed into their number-equivalent formulas, D0, D1, D2, these three create a profile that gives a very rich summary of diversity [5,30,31,32,33,34].

As pointed out above, Margalef [20,21] recognized the distinction between frequency-based and count-based diversity, and therefore he was very alive to the idea of “evenness”: the departure of diversity from the value that could be attained if all species were equally frequent [35]. Later, researchers formalized this idea into evenness indices such as “actual diversity” divided by “maximum attainable diversity” for a given S, either in the “D” scale [36,37] (p. 256ff) or for Réyni entropy [38].

Margalef positively bubbled with other ideas relating to diversity, information, and entropy, which led to many years of work by other scientists [12]. Even in these early papers [4,12,20,21], in addition to simply presenting new diversity measures, especially the entropy/information measure, Margalef was doing many things that nowadays remain very active areas of biodiversity research. He always searched for unifying principles to combine different aspects of ecology [39]. Margalef was likely the first to see the generality that information theory gives to biology, proposing that the whole of nature could be expressed as three channels of information: genetic, ecological, and behavioral [29] (p. 97, Figure 12). It was not until some years later that researchers began to look at the associations between these three channels, such as a possible relationship between the species diversity of an ecosystem and the genetic diversity within each species contained in that system [40,41].

Margalef’s attention to genetics and behavior [29] was also prophetic. However, we should note that he was not the first to note the application to genetics, where the honor goes to Shannon himself in an unpublished thesis [42] that was largely unknown. Margalef may have been alerted to genetic information by Branson’s application to proteins [16], which at the time were known to be genetically variable. In typical genetic applications, *p_i_* is the proportional abundance of the *i*th genetic variant in a population of *S* different individuals in one species [5]. Behavior is now also being quantified in information theory terms, so that the complexity of behavior between widely different species such as birds and humans can be compared and so that its fitness implications can be considered [43,44,45].

More recent uses of entropy in ecology have included assessments of spatial patterns. Maximum entropy has been used to assess species distributions [46] and to understand why biodiversity patterns can often be explained by theory that is neutral, i.e., that considers all species to have similar properties [47]. Maximum relative entropy has been used to unify, across all spatial scales, species abundance patterns in ecological communities [48]. Margalef noted that there seems to be an upper limit to diversity of approximately ~4.5 bits in the log 2 scale and puzzled over this in many papers [29] (p. 57) [49]. It was a decade and a half before his ideas on a limit to biodiversity were developed formally using maximum entropy principles [50].

Margalef’s interest in spatial aspects of diversity and the inherent sampling issues for Shannon diversity were discussed in many early papers, such as in Reference [29] (p. 55). First, he highlighted biologically important aspects, such as changes in diversity with increased extents of sampling, calling this the “diversity spectrum” and pointing out its likely connection to physical processes that affect diversity, such as ocean currents [22]. Second, there were statistical issues, because Equation (1) is sensitive to species (or alleles) approximately in proportion to their abundance, so that missing rare species can affect estimates [51] (p. 10) [52]. Margalef [23] (p. 109) speculated that relative abundances of the commoner species might be used to infer the abundances of the (missing) rare species. This pioneering idea has now been formalized by a method based on the work of another pioneer (in computing), Turing, and it does indeed use the relative abundances of the commoner species to infer the presence or absence of the rare ones [53,54].

## 3. Shannon Entropy and Information: Processes in Ecology

Shannon methods have not only been used for describing diversity patterns, but also for examining processes [55]. When considering processes, Margalef followed two main themes: adding functional interactions to diversity measures and considering ecosystem interactions as thermodynamic networks. Margalef’s early papers attempted to relate habitat characteristics to species in the diversity calculation, as noted above. Margalef summarized his thoughts on this, using the term “functional diversity of ecosystems” [23] (p. 115). He suggested that the dominance of a species should be assessed not via sheer numbers or biomass, but by net effects on other species, and that functional diversity could be expressed as a “connectance” coefficient derived from H2-type diversity [23] (p. 84):

$$Connectance=\sum a_{ij}p_{i}p_{j}/\sum p_{i}p_{j},$$

where $p_{i}p_{j}$ are the relative proportions of two species, and $a_{ij}$ is the intensity of their interaction. Alternatively, he noted that there was a possible H1-type Shannon-based formulation derived from Volterra’s [56] demonstration, where the effect of each species on others might be shown as $\left(mean\;effect\;of\;one\;individual\right)*N_{i}\;ln\;N_{i}$, which has a striking similarity to the Brioullin and Shannon formulae (Equations (1) and (3)) [23] (p. 110). However, it was a long time before functional diversity was satisfactorily incorporated into diversity measures without violating the desirable properties of a diversity measure [5,32], with this still being a vexed question in this century [41]. Modern methods are now available to incorporate functional traits into diversity measures, so that, for example, a community with species having similar traits is identified as being less diverse than a community with an identical abundance distribution but whose species have traits that diverge from one another considerably [6].

Margalef [23] (p. 110) was keen to assess interactions along all possible paths in a food web and proposed the use of Volterra’s [56] relationship to model the importance of diversity in successional processes. However, he pointed out the difficulty of assessing this along all possible paths in a food web and suggested that this challenge might be addressed by the method of Ulanowicz [57]. Similarly, Jorgensen [58] built on Margalef’s entropic ideas to incorporate the Kullback–Liebler formula, which is closely related to Shannon’s Equation (1), as a measure of the change in information due to increased knowledge of a part of the system. Margalef also attempted to link changes in abundance to changes in the number of species, suggesting that another index, k=lnS/lnN, would depend upon the relative rate of change in *S* and *N* and that each of these depends upon positive and negative feedback loops [23] (p. 112). Note that this index departs from Margalef’s earlier reliance on Gleason’s finding that in some cases, the number of species is linearly related to the log of the area sampled [13].

Margalef gave considerable thought to how to use diversity measures to systematize temporal succession and spatial turnover [29] (pp. 72–73). He produced extremely influential ideas about the interrelationship between diversity, succession, energy flows, productivity, and biomass, considering the thermodynamic implications of formulating diversity using entropic formulae. His interest and ideas about stability and resilience were reviewed by Prat [59]. Margalef suggested that the productivity to biomass ratio should have a negative correlation with diversity [29] (p. 66). He believed that any associations between biomass and productivity would not be direct, as some others were proposing, but would be through their rates of change (first or second order) [23] (p. 96) [29] (p. 22, Equations (6) and (7)). He also proposed that a good measure of “efficiency” in an ecosystem would be “information increase”/“entropy increase”, presumably to assess the way that order is attained at the expense of creating disorder elsewhere in the system [23] (p. 123). He also made proposals about the interdependence between the diversity and stability of ecosystems [60]. However, there were other theories, such as Odum’s, and by 1972, there was still little evidence to evaluate these theories [27] (p. 551). The relationship between the diversity and stability of ecosystems is still regarded as having great importance in basic science and conservation, but it remains contentious, and of course it depends on definitions such as “stability” and “productivity” [61,62,63,64]: Margalef took the latter to be “primary production per unit biomass” [23].

Entropy/information methods provide the added benefit that they can also be used to comprehensively characterize the physical world that interacts with biota [65,66,67,68].

## 4. Conclusions: Information, Entropy, and Margalef in Ecology and Evolution

Margalef’s introduction of Shannon–Weiner entropy into ecology and his enthusiastic championing of this measure have led to powerful methods for assessing both patterns and processes throughout ecology and evolution, which are now used enthusiastically and widely.

Throughout his life, Margalef was interested in entropy and information, especially the importance of thermodynamic irreversibility in ecosystem processes such as succession. In his later book, *Our Biosphere* [23], there are seven chapters that reflect on the significance of entropy for different aspects of life: energy, succession, diversity, dissipative/self-organizing systems, creative constraints, and evolution. Although clearly not at all afraid of mathematics, Margalef never seriously attempted to mathematically introduce entropy into ecology, which was done by some of his students and colleagues, which represents just a part of his continuing influence on ecology [69].

Margalef’s championing of these methods in ecology also led to their uptake in the related field of evolution, where Shannon methods are regarded as one of the best ways of summarizing adaptive change [66]. A recent attempt at unifying the whole of ecology and evolutionary genetics minimized the mathematical basis [70], but it has recently been suggested that an entropy/information approach is ideal for this unification [5,65,66,67,68].

## References

[1] Prat N., Ros J., Peters F. (2015). Ramon Margalef, Ecólogo de la Biosfera una Biografía Científica.

[2] Catalunya G.D. (2019). The Premi Ramon Margalef D’Ecologia. https://presidencia.gencat.cat/ca/ambits_d_actuacio/premis/premi-ramon-margalef-decologia/.

[3] (2019). Entropy. https://www.mdpi.com/journal/entropy/special_issues.

[4] Margalef R. (1949). Una Aplicacion de las Series Logaritmicas a la Fitosociolgia.

[5] Sherwin W., Chao A., Jost L., Smouse P. (2017). Information Theory Broadens the Spectrum of Molecular Ecology and Evolution. Trends Ecol. Evol..

[6] Chao A., Chiu C.-H., Villéger S., Sun I.-F., Thorn S., Lin Y.-C., Chiang J.-M., Sherwin W.B. (2019). An attribute-diversity approach to functional diversity, functional beta diversity, and related (dis)similarity measures. Ecol. Monogr..

[7] Fisher R.A., Corbet A.S., Williams C.B. (1943). The Relation Between the Number of Species and the Number of Individuals in a Random Sample of an Animal Population. J. Anim. Ecol..

[8] Preston F.W. (1948). The commonness, and rarity, of species. Ecology.

[9] Whittaker R.H. (1960). Vegetation of the Siskiyou Mountains, Oregon and California. Ecol. Monogr..

[10] Shannon C.E., Weaver W. (1949). The Mathematical Theory of Communication.

[11] Cover T.M., Thomas J.A. (1991). Elements of Information Theory.

[12] Margalef R. (1951). Diversidad de Especies en las Comunidades Naturales. Publ. Inst. Biol. Apl..

[13] Gleason H.A. (1922). On the Relation Between Species and Area. Ecology.

[14] Margalef R. (1955). La diversidad de especies en las poblaciones mixtas naturales y en el estudio y dinamismo de las mismas. Tomo de Homenaje Póstumo al Dr. Francisco Pradillo Vaquer.

[15] Margalef R. (1956). Información y diversidad específica en las comunidades de organismos. Investig. Pesq..

[16] Branson H.R., Quastler R. (1953). Information Theory and the Structure of Proteins. Information Theory in Biology.

[17] Wiener N. (1948). Cybernetics.

[18] Zeng G. (2015). A Unified Definition of Mutual Information with Applications in Machine Learning. Math. Probl. Eng..

[19] Shannon C.E. (1948). A Mathematical Theory of Communication. Bell Syst. Tech. J..

[20] Margalef R. (1957). La Teoría de la Información en Ecología. Mem. Real Acad. Cienc. Artes Barc..

[21] Margalef R. (1958). Information theory in ecology. Gen. Syst..

[22] Estrada M., Valladares F. (2008). Explorations on Phytoplankton Diversity. An Appreciation of Ramon Margalef’s Contributions. Unity in Diversity. Reflections on Ecology after the Legacy of Ramon Margalef.

[23] Margalef R. (1997). Our Biosphere. Excellence in Ecology, 10th, ed..

[24] Peters F. (2010). Ramon Margalef, the Curiosity Driven Life of a Self-Taught Naturalist.

[25] Buddle C.M., Beguin J., Bolduc E., Mercado A., Sackett T.E., Selby R.D., Varady-Szabo H., Zeran R.M. (2004). The importance and use of taxon sampling curves for comparative biodiversity research with forest arthropod assemblages. Can. Entomol..

[26] Pielou E. (1966). The measurement of diversity in different types of biological collections. J. Theor. Boil..

[27] Krebs C.J. (1972). Ecology: The Experimental Analyisis of Distribution and Abundance.

[28] Wilhm J.L. (1968). Use of Biomass Units in Shannon’s Formula. Ecology.

[29] Margalef R. (1968). Perspectives in Ecological Theory.

[30] Chao A., Chiu C.-H., Jost L. (2014). Unifying species diversity, phylogenetic diversity, functional diversity, and related similarity and differentiation measures through Hill numbers. Annu. Rev. Ecol. Evol. Syst..

[31] Pielou E.C. (1974). Population and Community Ecology: Principles and Methods.

[32] Leinster T., Cobbold C.A. (2012). Measuring diversity: The importance of species similarity. Ecology.

[33] Hill M.O. (1973). Diversityand evenness: A unifying notation and its consequences. Ecology.

[34] Marcon E., Scotti I., Hérault B., Rossi V., Lang G. (2014). Generalization of the Partitioning of Shannon Diversity. PLoS ONE.

[35] Peet R.K. (1974). The Measurement of Species Diversity. Annu. Rev. Ecol. Syst..

[36] Lloyd M., Ghelardi R.J. (1964). A Table for Calculating the ‘Equitability’ Component of Species Diversity. J. Anim. Ecol..

[37] Legendre P., Legendre L. (2012). Numerical Ecology.

[38] Jost L. (2010). The Relation between Evenness and Diversity. Diversity.

[39] Margalef R. (1963). On Certain Unifying Principles in Ecology. Am. Nat..

[40] Vellend M. (2005). Species Diversity and Genetic Diversity: Parallel Processes and Correlated Patterns. Am. Nat..

[41] Magurran A.E. (2004). Measuring Biological Diversity.

[42] Shannon C.E. (1936). An Algebra for Theoretical Genetics.

[43] Hailman J.P., Ficken M.S., Ficken R.W. (1985). The ‘chick-a-dee’ calls of Parus atricapillus: A recombinant system of animal communication compared with written English. Semiotica.

[44] Bergstrom C.T., Donaldson-Matasci M.C., Lachmann M. (2005). The fitness value of information. arXiv.

[45] Lachmann M., Bergstrom C.T. (2004). The disadvantage of combinatorial communication. Proc. R. Soc. B Boil. Sci..

[46] Elith J., Phillips S.J., Hastie T., Dudík M., Chee Y.E., Yates C.J. (2011). A statistical explanation of MaxEnt for ecologists. Divers. Distrib..

[47] Pueyo S., He F., Zillio T. (2007). The maximum entropy formalism and the idiosyncratic theory of biodiversity. Ecol. Lett..

[48] Dewar R.C., Porté A. (2008). Statistical mechanics unifies different ecological patterns. J. Theor. Boil..

[49] Margalef R. (1972). Homage to Evelyn Hutchinson, or Why There Is an Upper Limit to Diversity.

[50] Wagensberg J., Lopez D., Valls J. (1988). Statistical aspects of biological organisation. J. Phys. Chem. Solids.

[51] Pielou E.C. (1975). Ecological Diversity.

[52] Gamito S. (2010). Caution is needed when applying Margalef diversity index. Ecol. Indic..

[53] Chao A., Chiu C.-H., Colwell R.K., Magnago L.F.S., Chazdon R.L., Gotelli N.J., Colwell R.K., Magnago L.F.S., Chazdon R.L., Gotelli N.J. (2017). Deciphering the enigma of undetected species, phylogenetic, and functional diversity based on Good-Turing theory. Ecology.

[54] Chao A., Jost L. (2015). Estimating diversity and entropy profiles via discovery rates of new species. Methods Ecol. Evol..

[55] Macarthur R. (1955). Fluctuations of animal populations and a measure of community stability. Ecology.

[56] Volterra V. (1937). Principes de biologie mathematique. Acta Biotheor..

[57] Ulanowicz R.E. (1986). Growth and Development: Ecosystems Phenomenology.

[58] Jorgensen S.E., Valladares F. (2008). Towards an Ecosystem Theory. Unity in Diversity. Reflections on Ecology after the Legacy of Ramon Margalef.

[59] Prat N. (2015). Stability, resilience and sustainability: A tribute to Ramon Margalef, 10 years after his death. Limnetica.

[60] Margalef R. (1969). Diversity and stability: A practical proposal and a model of interdependence. Brookhaven Symp. Boil..

[61] Chiarucci A., Bacaro G., Scheiner S.M. (2011). Old and new challenges in using species diversity for assessing biodiversity. Philos. Trans. R. Soc. B Boil. Sci..

[62] Conti G., Díaz S. (2013). Plant functional diversity and carbon storage—An empirical test in semi-arid forest ecosystems. J. Ecol..

[63] Gagic V., Bartomeus I., Jonsson T., Taylor A., Winqvist C., Fischer C., Slade E.M., Steffan-Dewenter I., Emmerson M., Potts S.G. (2015). Functional identity and diversity of animals predict ecosystem functioning better than species-based indices. Proc. R. Soc. B Boil. Sci..

[64] Lefcheck J., Duffy J.E. (2015). Multitrophic Functional Diversity Predicts Ecosystem Functioning in Experimental Assemblages of Estuarine Consumers. Ecology.

[65] Day T. (2015). Information entropy as a measure of genetic diversity and evolvability in colonization. Mol. Ecol..

[66] Frank S.A. (2017). Universal expressions of population change by the Price equation: Natural selection, information, and maximum entropy production. Ecol. Evol..

[67] Frank S.A. (2018). The Price Equation Program: Simple Invariances Unify Population Dynamics, Thermodynamics, Probability, Information and Inference. Entropy.

[68] Sherwin W.B. (2018). Entropy, or Information, Unifies Ecology and Evolution and Beyond. Entropy.

[69] Valladares F., Camacho A., Elosegi A., Gracia C. (2018). Reflections on Ecology after the Legacy of Ramon Margalef.

[70] Vellend M. (2016). The Theory of Ecological Communities (MPB-57).

